# Supplementary material for: Effect of methylene blue on experimental postoperative adhesion: A systematic review and meta-analysis
Source: PLoS One. 2022 May 19;17(5):e0268178. doi: 10.1371/journal.pone.0268178 (PMC9119438; doi:10.1371/journal.pone.0268178)
Supplement: S1 File — (DOCX) [file pone.0268178.s002.docx]

***Appendix***

**MEDLINE**

1. Exp General Surgery/
2. surgery.mp.
3. opertation.mp
4. opera$.mp
5. surg$.mp
6. or/1-5
7. exp Tissue Adhesions/
8. adhesion.mp
9. adhesive.mp
10. adhesi$.ab,ti.
11. or/7-10
12. 6 and 11
13. exp Methylene Blue/
14. Methylthionin$.mp
15. Urolene.mp
16. Urelene.mp
17. Provayblue.mp
18. Proveblue.mp
19. Swiss Blue.mp
20. [CI](https://en.wikipedia.org/wiki/Colour_Index_International) 52015.mp
21. basic blue 9.mp
22. or/13-21
23. 12 and 22

**EMBASE**

1. 'general surgery'/exp
2. surgery
3. operation
4. surg$
5. operat$
6. #1 OR #2 OR #3 OR #4 OR #5
7. #1 OR #2 OR #3 OR #4 OR #5 AND [embase]/lim
8. 'adhesion'/exp
9. Adhesion
10. 'adhesion barrier'/exp
11. adhesi$.
12. #8 OR #9 OR #10 OR #11
13. #7 AND #12
14. ‘Methylene Blue’/ exp
15. Methylthionin$
16. Urolene
17. Urelene
18. Provayblue
19. Proveblue
20. Swiss AND Blue
21. [CI](https://en.wikipedia.org/wiki/Colour_Index_International) 52015.mp
22. Basic AND blue AND 9
23. #14 OR #15 OR #16 OR #17 OR #18 OR #19 OR #20 OR #21 OR #22
24. #13 AND #23

**Figure legend for supplementary figure**


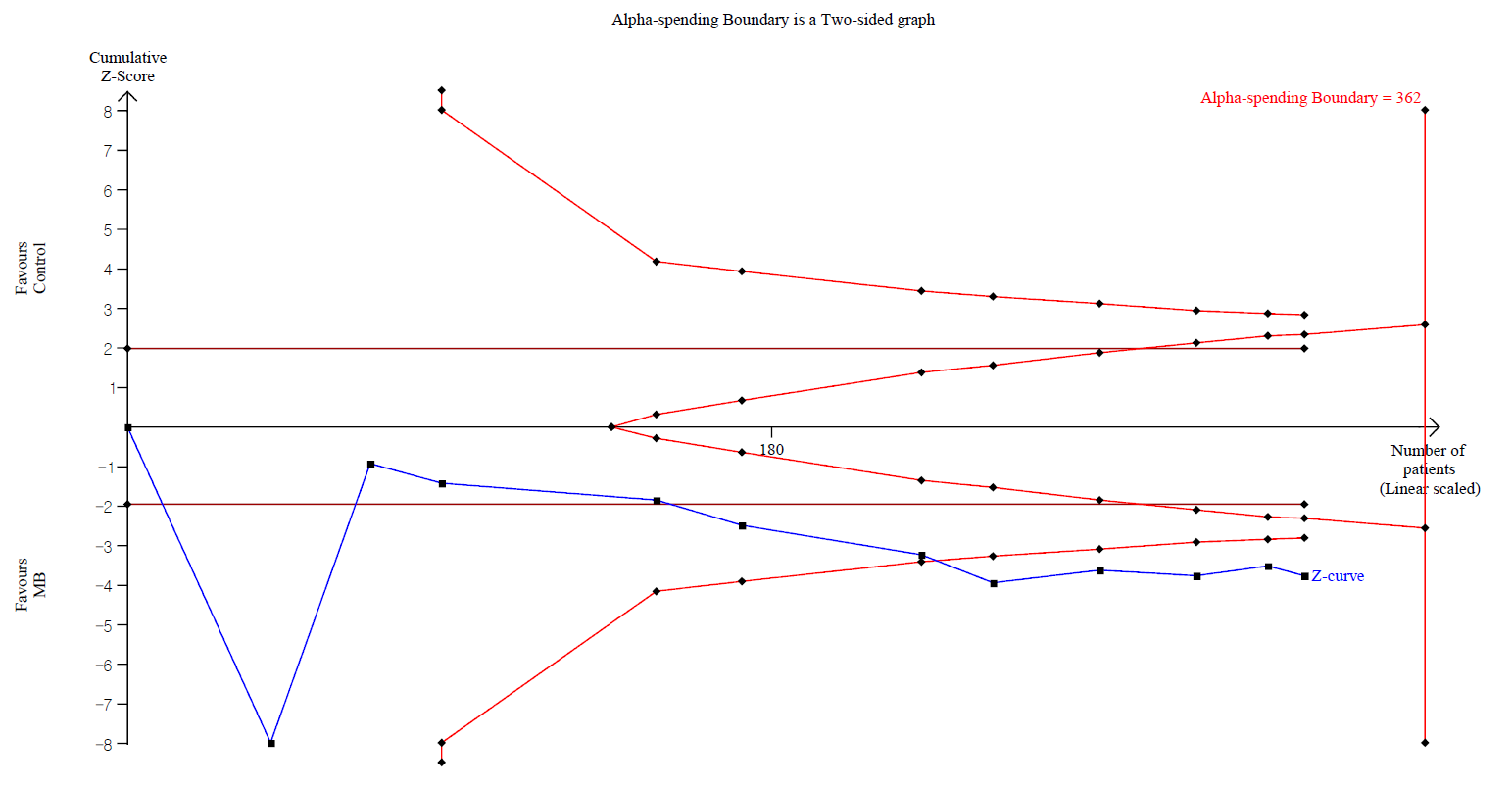


**Supplementary figure 1. Trial sequence analysis for macroscopic adhesion score.** The cumulative Z curve (complete blue curve, ) crossed both the conventional test boundary (etched red line, ) and the trial sequential monitoring boundary (complete red curve, )
