# Supplementary material for: Effect of methylene blue on experimental postoperative adhesion: A systematic review and meta-analysis
Source: PLoS One. 2022 May 19;17(5):e0268178. doi: 10.1371/journal.pone.0268178 (PMC9119438; doi:10.1371/journal.pone.0268178)
Supplement: Attachment — Submitted filename: Response to reviewers letter.docx [file pone.0268178.s004.docx]

**Dear Yuan-Pin HsuAcademic Editor, *PLOS ONE*,**

We thank sincerely Editor and Reviewers of the ‘***PLOS ONE’*** for taking their precious time to review our paper. Your constructive, meticulous and considerate comments were great guidance for our aforementioned manuscript. According to your precious comments and suggestions, we sincerely and earnestly tried to response for your letter. We want to express my heartfelt gratitude for your comments once more.

We have made some corrections in the manuscript after going over your comments. We highlighted the modification made to the original document by using red colored text. The changes are summarized below:

**Reviewer #1: Summary

The authors have performed a systematic review and meta-analysis of the literature on the effects of methylene blue on post-operative adhesions in animal models.

Comments
This work is original and informative. While meta-analyses can be found on human studies, I was not able to find the equivalent in experimental models.
It would possibly add to the value of the study if the authors expanded on any reported side effects of the methylene blue treatment from the selected manuscripts.**

**Our response:** Thank you for reviewer’s support and suggestion. We agree with reviewer’s comment. According to reviewer’s comment, we reviewed all studies included in this systematic review and meta-analysis for the side effects of methylene blue. However, all the studies included did not report any side effects of methylene blue. We describe it in the manuscript **(Methods section 1^st^ paragraph on page 8, Results section 2nd paragraph on page 25)**

**While the manuscript is presented in an intelligible fashion, there are some grammatical errors.**

**Our response:** Thank you for reviewer’s comment. According to reviewer’s comment, we.re-performed English editing from editage and Job code is ACUNE_7542_2.

**Reviewer #2: the authors are presenting the first meta-analysis for the effect of Methylene Blue on post-operative intra-abdominal adhesions. The authors implemented that no language exclusion was applied. I was wondering if in the case of a manuscript that is written in Spanish or other language that the authors are not familiar with, what was the situation and if that did not happen, I believe it should be mentioned in the methods section.**

**Our response:** Thank you for reviewer’s comment. We planned to consult and co-work with experts affiliated with our university, when foreign language translation was necessary.

In the first stage of study selection, namely when study selection was performed from the title or abstract, full text of some articles were written in many other languages. But title and abstract of these articles are written in English. Therefore, we can perform first stage of study selection.

And in the second stage of study selection, namely when full text versions were evaluated, all the articles were written in English. Therefore, we did not consult and co-work with experts affiliated.

According to reviewer’s comment, we described this in the manuscript. **(Methods section 1st paragraph on page 6, Results section 1st paragraph on page 11)**

**Some repetitive sentences were encountered when explaining the method used to resolve conflict between the two authors responsible for the literature search / review. It would be of significance if these sentences were altered.**

**Our response:** Thank you for reviewer’s comment. According to reviewer’s comment, we altered these sentences. **(Methods section last paragraph on page 6,)**

**Some minor grammatical errors were found that can be easily fixed with a simple revision.**

**Our response:** Thank you for reviewer’s comment. According to reviewer’s comment, we.re-performed English editing from editage and Job code is ACUNE_7542_2.

**Reviewer #3: The author, in the limitation, mentioned that there is significant heterogeneity in the studies due to the different surgical approaches. Could performing a subgroup analysis based on surgical procedures be helpful to the readers?**

**Our response:** Thank you for reviewer’s suggestion for the betterment of the manuscript. According to reviewer’s comment, we performed subgroup analysis based on surgical procedures. However, the heterogeneity was not decreased. We added the description for subgroup analysis based on surgical procedures in the manuscript. **(Methods section last paragraph on page 8, Results section 2^nd^, 3^rd^ paragraph on page 22, 1^st^ paragraph on page 23, Discussion section 2^nd^ paragraph on page 30)**

**The adhesion grading shown in table 2 is not uniform among the studies. How did the author attempt to unify the values to calculate a pooled estimate?**

**Our response:** Thank you for reviewer’s comment. As reviewer recommended, the results in table 2 is not uniform among the studies. As described in results section on page 13, studies used 5 point scale, 4 point scale, 5 and 6 point scale, cumulative scale or percentage. All these scales showed the severity of macroscopic adhesion score. Therefore, as described in the manuscript, we used the standardized mean difference to calculate a pooled estimate. The **standardized mean difference** is commonly used as a summary statistic in meta-analysis when the studies all assess the same outcome but measure it in a variety of ways

We hope the revised manuscript will better meet the requirements of the ‘***PLOS ONE***’ for publication. Again, we are most grateful for the constructive review by Editor and reviewers of the ‘***PLOS ONE’***.

Sincerely yours,

**Hyun Kang, MD, PhD, MPH**

**Professor,**

**Department of Anesthesiology and Pain Medicine,**

**Chung-Ang University College of Medicine,**

**84 Heukseok-ro, Dongjak-gu, Seoul, 06911, Republic of Korea.**

**Tel: +82-2-6299-2571, 2579, 2586; Fax: +82-2-6299-2585**

**E-mail:** [roman00@naver.com](mailto:roman00@naver.com)
